# Text-Book on Mental Diseases

**Published:** 1897-11

**Authors:** 


Text-Book on Mental Diseases. By Theo. H. Kellogg, A. M., M. D., Late Medical Superintendent of Willard State Hospital, etc., etc. Octavo, 792 pages; illustrated by original sphygmographic tracings and photographs of the different types of mental disorder. New York: William Wood & Co. 1897.

The appearance of this superb and exhaustive work is an honor to American psychiatry and a credit to the author who stands in the front rank of medico-psychologists. It embraces the results of many years' study in the various hospitals for the insane in which the author has been engaged and shows his skill and aptitude in dealing with perhaps the most abstract and subtle of all the medical sciences.

The author divides his work into two parts: Part I. dealing with general mental pathology, and Part II. with the special groups and the typical forms of insanity.

The chapters comprising Part I. treat of the history of insanity from the dawn of the Greek school of medicine to the present time—the statistics, nosology, etiology, the evolutions, stadia, clinical progression and termination of mental disorders, the psychical and somatic symptomatology, pathology, diagnosis, prognosis and treatment of insanity.

The author's classification of the insanities, on page 60, embraces all the older recognised and some of the new forms and is divided into two groups. Group A (etio-pathological) embraces the insanities with definitely assignable etiological and pathological relations, while group B (psycho-symptomatological) embraces those without definitely assignable etiological and pathological relations or, in other words, are the so-called psychoses, such as the manias, melancholias and dementias. Interesting in the classification is the number of toxic and diathetic insanities. A few years ago these insanities, arising from general systemic morbid states, were very few, limited almost entirely to alcoholism and morphinism; now, however, plumbism, hydrargyrism, oxycarbonism, cocainism, bromidism, etherism, chloroformism, chloralism, nicotinism, autointoxications, lyssa humana and infectious diseases must be included in the list, while under the diathetic the author includes the phthisical, cancerous, podagrous, rheumatic, pellagrous, limopsoitosis, malarious, anemic, post-febrile and myxedematous forms. These classes are more etiological than separate forms of insanity, otherwise it would tend to confuse the status of insanity-expertism more than ever. The chapters in Part I. are intensely interesting and many important and suggestive facts are brought out, which cannot find place in a cursory review.

Part II. treats in the same entertaining way of the old recognised forms of insanity, and although but little new has been added to our conception of these forms, yet the author dresses them in a pleasing and attractive style, making them appear like new. The whole subject is brought down to the very latest time, and although remembering all the newer ideas and forms of treatment, still the author does not allow these novelties to overshadow the accustomed standbys.

William Wood & Co. are the publishers, which is guarantee enough of the make-up of the book. W. C. K.

